# Association of SII and SIRI with incidence of cardiovascular disease in cardiovascular-kidney-metabolic syndrome: a prospective cohort study

**DOI:** 10.3389/fnut.2025.1661826

**Published:** 2025-11-24

**Authors:** Chen Pengfei, Megan Lui, Lixiao Zhang, Chen Chen, Tianyu Wang, Hou Ailin, Deng Pan, Wenxi Yu, Jianpeng Du, Ming Guo, Dazhuo Shi

**Affiliations:** 1 Xiyuan Hospital, China Academy of Chinese Medical Sciences, Beijing, China; 2 Department of Population Health Sciences, Weill Cornell Medical College, New York, NY, United States; 3 Hangzhou Traditional Chinese Medicine Hospital, Hangzhou, China

**Keywords:** cardiovascular disease incidence, cardiovascular-kidney-metabolic syndrome, systemic immune-inflammation index, systemic inflammation response index, UK biobank

## Abstract

**Background:**

The relationship between systemic immune-inflammation biomarkers, the Systemic Immune-Inflammation Index (SII) and the Systemic Inflammation Response Index (SIRI), and cardiovascular disease (CVD) in cardiovascular-kidney-metabolic (CKM) syndrome remains unclear.

**Methods:**

Individuals with CKM syndrome at stages 0–3 from UK Biobank were included in this study. Cox regression models were used to assess the association of SII and SIRI with incidence of CVD. Restricted cubic spline (RCS) models were applied to examine nonlinear longitudinal associations. Subgroup stratified by CKM stages were analyzed.

**Results:**

A total of 301,631 participants with CKM stages 0 (14.1%), 1 (19.4%), 2 (56.7%), and 3 (9.8%) were included in this study. During a median follow-up of 13.0 years, CVD incidence occurred in 35,782 (11.86%) participants. After adjustment, the elevated SII (HR: 1.07, 95% CI: 1.05–1.10, *p* < 0.001) and SIRI (HR: 1.31, 95% CI: 1.26–1.37, *p* < 0.001) were associated with the risk of CVD incidence. RCS analyses indicated a U-shaped association for SII and a monotonic increase for SIRI. Subgroup analyses revealed significantly stronger associations between both SII and SIRI and the incidence of CVD with CKM stages.

**Conclusion:**

As the main systemic immune-inflammation biomarkers, the elevated SII and SIRI in CKM syndrome were significantly associated with increased risk of CVD incidence.

## 1 Introduction

Cardiovascular-kidney-metabolic (CKM) syndrome is a systemic disorder characterized by pathophysiological interactions among metabolic risk factors, chronic kidney disease (CKD), and cardiovascular system, which lead to a high incidence of cardiovascular diseases (CVD) (1). The progression of CKM syndrome is linked to chronic low-grade inflammation and immune dysfunction (2, 3).

The systemic immune-inflammation index (SII) and systemic inflammation response index (SIRI) have been recognized for over a decade as key biomarkers of systemic immune-inflammation in cardiovascular research (4). These markers incorporate neutrophils, monocytes, lymphocytes, and platelets, and reflect the complex interactions among innate immunity, adaptive immune suppression, and prothrombotic states (5).

The latest studies indicate that individuals with systemic immune-inflammation conditions have a high incidence of CVD as compared to healthy populations, highlighting the high immune-inflammation as the potential predictive indicators for CVD (6, 7). Although SII and SIRI are the established biomarkers of immune-inflammation, their association with CVD incidence among individuals with CKM syndrome remains unclear. To address this gap, this prospective study was conducted using data from the UK Biobank (UKB).

## 2 Methods

### 2.1 Study population

This study recruited 392,965 CKM population of UKB, at stage 0 to 3, aged 40–69 years. Baseline characteristics were derived from the initial assessment visit (Instance 0, 2006–2010), including demographic characteristics, lifestyle factors, medical history, medication, anthropometric measurements, and biochemical markers. All participants provided informed consent for participation and follow-up (8).

**Figure 1:**
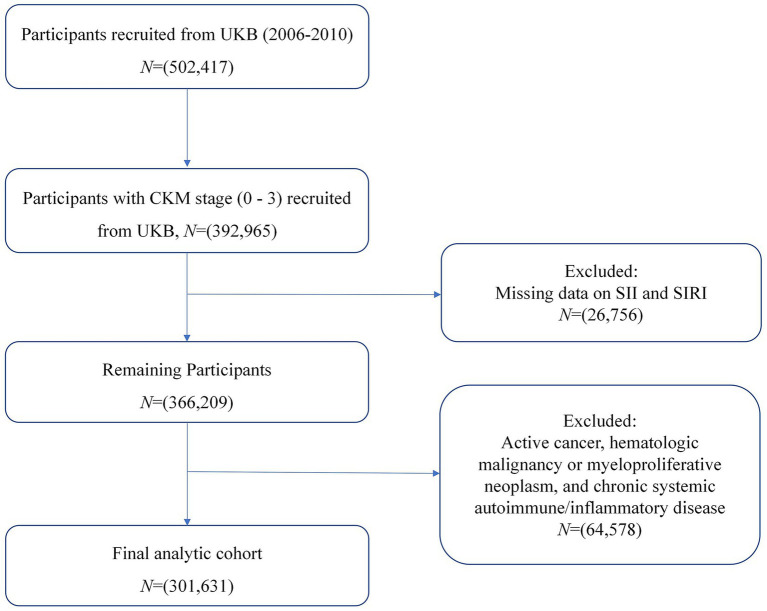
Flowchart of study participants.

The exclusion criteria were as follows: (1) missing data for SII and SIRI (*n* = 26,756); (2) active cancer, hematologic malignancy or myeloproliferative neoplasm (leukemia, lymphoma, multiple myeloma, essential thrombocythemia), and chronic systemic autoimmune/inflammatory disease (rheumatoid arthritis, ankylosing spondylitis, systemic lupus erythematosus, systemic vasculitides, multiple sclerosis) (*n* = 64,578) (Figure 1).

### 2.2 Calculation of SII and SIRI

In the UKB, blood specimens were obtained from participants, underwent minimal local processing, and were subsequently transferred to a central facility for storage and further analysis. Standard hematology assays were conducted within 24 h using the Beckman Coulter LH750 analyzer.

The calculation formulas for SII and SIRI were as follows: SII was calculated as platelet count × neutrophil count/lymphocyte count, while SIRI was defined as neutrophil count × monocyte count/lymphocyte count. All units were expressed as 10^9^/L (5, 9).

### 2.3 Incidence of CVD

Outcomes were ascertained using ICD-10 codes from hospital inpatient records and death registries. The primary outcome was CVD, defined as CHD (ICD-10 codes I20–I25), AF (I48), HF (I50), or CBVD (I60–I64, I67–I69) (10). Time-to-event was measured from baseline to whichever occurred first: CVD diagnosis, death, or censoring (as of 31 December 2022).

### 2.4 Definition of CKM syndrome stages 0–3

CKM syndrome and its stages were diagnosed based on the framework outlined in the AHA’s Presidential Advisory Statement on CKM Syndrome (1). The stages are divided as follows: Stage 0 is characterized by normal weight, blood glucose, blood pressure, lipid levels, and kidney function. Stage 1 is characterized by obesity or adiposity dysfunction, prediabetes or other early metabolic disturbances. Stage 2 involves individuals with established metabolic risk factors, such as type 2 diabetes, hypertension, elevated triglycerides, or moderate kidney dysfunction. Stage 3 includes subclinical cardiovascular disease. In this classification, very high-risk chronic kidney disease (stage G4 or G5) and a high predicted 10-year cardiovascular disease risk, based on the Framingham risk score, are considered equivalent to subclinical CVD (11, 12). The specific criterion of CKM stage was annotated in Supplementary Table 1.

### 2.5 Assessment of covariates

Demographic covariates included age, sex, ethnicity, Townsend Deprivation Index (greater values signifying more pronounced socioeconomic disadvantage), and education level. Lifestyle factors included sleep duration, smoking status, alcohol status, and physical activity. Clinical and laboratory variables included body mass index (BMI, kg/m^2^), hypertension status, diabetes status, high-density lipoprotein cholesterol (HDL-C, mmol/L), low-density lipoprotein cholesterol (LDL-C, mmol/L), triglycerides (TG, mmol/L), total cholesterol (TC, mmol/L), high-sensitivity C-reactive protein (hs-CRP, mg/L), serum uric acid (SUA, μmol/L), serum creatinine (SC, μmol/L), glucose (mmol/L), and estimated glomerular filtration rate (eGFR, calculated using the CKD-EPI 2009 creatinine equation and expressed in mL/min/1.73m^2^).

### 2.6 Statistical analysis

Baseline characteristics were compared using independent t-tests or Mann–Whitney U tests for continuous variables and chi-square tests for categorical variables. Continuous variables were expressed as mean ± SD or median (IQR), and categorical variables as *n* (%). The multivariable Cox proportional hazards regression was applied to estimate hazard ratios (HRs) and 95% confidence intervals (CIs) (13). The proportional hazards assumption was verified via Schoenfeld residuals. Log-transformed SII and SIRI were assessed both continuously (per 1-unit increase) and categorically (quartiles, Q1–Q4). Model 1 adjusted for age and sex; Model 2 additionally included ethnicity, socioeconomic status, education, alcohol use, smoking, sleep status, and physical activity; and Model 3 further adjusted for metabolic and clinical variables (diabetes, hypertension, BMI, LDL-C, HDL-C, eGFR, and hs-CRP). Multicollinearity was evaluated through variance inflation factors, with all values below 5, indicating no major issues with multicollinearity (Supplementary Table 2).

Dose–response relationships were examined using restricted cubic spline (RCS) models with four knots. To assess non-linear associations and identify inflection points, we used a two-stage Cox regression model with RCS. We further performed stratified RCS analyses by sex, hypertension, and diabetes status. Subgroup analyses were conducted according to age (<60 vs. ≥ 60 years), sex (male vs. female), ethnicity (white vs. non-white), education level (college/university, other qualifications, or no qualifications), smoking (current, former, or never), alcohol consumption (daily, 3–4 times/week, 1–2 times/week, 1–3 times/month, special occasions, or never), physical activity level (<600, 600–3,000, or >3,000 MET-minutes/week), BMI (<25 vs. ≥ 25 kg/m^2^), diabetes (yes vs. no), hypertension (yes vs. no), and CKM stage (stages 0, 1, 2, or 3). Mediation analysis was conducted to evaluate whether diabetes, hypertension, obesity, and Hyperuricemia mediated the associations of SII and SIRI with incidence of CVD. Nonparametric bootstrapping with 1,000 simulations estimated the causal mediation effects. To assess incremental predictive value, SII or SIRI was added separately to Model 3 Cox models, and receiver operating characteristic (ROC) curves with area under curve (AUC) were used to compare their discriminative performance for incident CVD. To validate the stability of our findings, sensitivity analyses was performed: first, participants with <2 years of follow-up were excluded to minimize reverse causality; second, propensity score matching (PSM) was applied to balance covariates between groups, enhancing the reliability of association estimates; third, age was used as the timescale instead of follow-up duration to mitigate residual confounding by age; and finally, Finally, to reduce overadjustment from overlap with CKM stage definitions, we repeated the analyses excluding diabetes, hypertension, BMI, LDL-C, HDL-C, and eGFR. All statistical analyses were conducted using R version 4.3.3 (R Foundation for Statistical Computing, Vienna, Austria), and a *p*-value < 0.05 was considered statistically significant.

## 3 Results

### 3.1 Baseline characteristics

This study included 301,631 participants with CKM stages 0 (42,530; 14.1%), 1 (58,516; 19.4%), 2 (171,024; 56.7%), and 3 (29,561; 9.8%). The mean age was 54.86 ± 8.12 years, and 170,708 (56.6%) were female. A total of 15,237 (5.1%) have diabetes, and 117,941 (39.1%) had hypertension. The participants with CVD were generally older, male, and had lower socioeconomic and educational levels, as compared with those without CVD (Table 1).

**Table 1:** Baseline characteristics of study participants stratified by CVD events.

Characteristic	*N* = 301,631	Cardiovascular diseases (35,782)	No cardiovascular diseases (265,849)	*p*-value
Age (years)	54.86 ± 8.12	59.38 ± 7.20	54.25 ± 8.05	<0.001
Sex, *n* (%)				<0.001
Female	170,708(56.6%)	14,835(41.5%)	155,873(58.6%)	
Male	130,923(43.4%)	20,947(58.5%)	109,976(41.4%)	
Race, *n* (%)				<0.001
White	284,956(94.5%)	34,260(95.7%)	250,696(94.3%)	
Not White	16,675 (5.5%)	1,522 (4.3%)	15,153 (5.7%)	
Education, *n* (%)				<0.001
College/university degree	142,851 (47.4%)	13,677 (38.2%)	129,174 (48.6%)	
Other qualification	84,967 (28.2%)	9,370 (26.2%)	75,597 (28.4%)	
No qualification	32,785 (10.9%)	4,865 (13.6%)	27,920 (10.5%)	
Unknown	41,028 (13.6%)	7,870 (22.0%)	33,158 (12.5%)	
Alcohol consumption, *n* (%)				<0.001
Daily	60,266 (20.0%)	7,985 (22.3%)	52,281 (19.7%)	
3–4 times/week	72,543 (24.1%)	7,988 (22.3%)	64,555 (24.3%)	
1–2 times/week	80,329 (26.7%)	8,981 (25.1%)	71,348 (26.9%)	
1–3 times/month	34,371 (11.4%)	3,659 (10.2%)	30,712 (11.6%)	
Special occasions	32,178 (10.7%)	4,123 (11.5%)	28,055 (10.6%)	
Never	21,735 (7.2%)	3,012 (8.4%)	18,723 (7.0%)	
Physical activity (MET-minutes/week)				<0.001
<600	41,097 (13.6%)	4,851 (13.6%)	36,246 (13.6%)	
600–3,000	185,181 (61.4%)	16,232 (60.4%)	168,949 (61.6%)	
>3,000	75,353 (25.0%)	9,315 (26.0%)	66,038 (24.8%)	
Smoke, *n* (%)				<0.001
Never	173,449 (57.5%)	17,368 (48.5%)	156,081 (58.7%)	
Former	95,625 (31.7%)	13,061 (36.5%)	82,564 (31.1%)	
Current	32,557 (10.8%)	5,353 (15.0%)	27,204 (10.2%)	
Diabetes, *n* (%)	15,237 (5.1%)	3,227 (9.0%)	12,010 (4.5%)	<0.001
Hypertension, *n* (%)	117,941 (39.1%)	18,637 (52.1%)	99,304 (37.4%)	<0.001
CKM stages, *n* (%)				<0.001
0	42,530 (14.1%)	759 (2.12%)	41,771 (15.7%)	
1	58,516 (19.4%)	1,407 (3.93%)	57,109 (21.5%)	
2	171,024 (56.7%)	13,624 (38.1%)	157,400 (59.2%)	
3	29,561 (9.8%)	19,992 (55.87%)	9,569 (3.60%)	
Townsend Deprivation Index	−1.43 ± 3.01	−1.29 ± 3.11	−1.45 ± 3.00	<0.001
Sleep duration (hours/day)	7.15 ± 1.03	7.15 ± 1.11	7.15 ± 1.01	0.99
BMI, kg/m^2^	26.61 ± 4.35	27.45 ± 4.56	26.50 ± 4.31	<0.001
Lymphocyte, ×10⁹ cells/L	1.94 ± 0.72	1.96 ± 0.75	1.93 ± 0.72	<0.001
Monocyte, ×10⁹ cells/L	0.46 ± 0.21	0.49 ± 0.28	0.46 ± 0.19	<0.001
Neutrophil, ×10⁹ cells/L	4.12 ± 1.37	4.29 ± 1.45	4.10 ± 1.35	<0.001
Platelet, ×10⁹ cells/L	253.88 ± 58.64	249.64 ± 60.44	254.45 ± 58.37	<0.001
SII	586.73 ± 335.95	601.66 ± 404.16	584.72 ± 325.63	<0.001
SIRI	1.06 ± 0.94	1.18 ± 1.55	1.04 ± 0.82	<0.001
HDL-C, mmol/L	1.48 ± 0.36	1.41 ± 0.35	1.48 ± 0.36	<0.001
LDL-C, mmol/L	3.65 ± 0.81	3.74 ± 0.84	3.64 ± 0.81	<0.001
TG, mmol/L	1.66 ± 0.97	1.85 ± 1.07	1.63 ± 0.96	<0.001
TC, mmol/L	5.83 ± 1.06	5.90 ± 1.10	5.82 ± 1.05	<0.001
SUA, μmol/L	297.18 ± 74.22	316.58 ± 75.76	294.57 ± 73.62	<0.001
Sr, μmol/L	70.91 ± 13.73	73.44 ± 16.14	70.57 ± 13.33	<0.001
hs-CRP, mg/L	2.25 ± 3.88	2.80 ± 4.68	2.17 ± 3.76	<0.001
Fasting Glucose, mmol/L	4.99 ± 0.96	5.10 ± 1.24	4.97 ± 0.91	<0.001
eGFR, mL/min/1.73 m^2^	94.18 ± 12.25	91.97 ± 13.36	94.76 ± 13.19	<0.001
DBP, mmHg	80.68 ± 9.89	82.44 ± 10.18	80.45 ± 9.83	<0.001
SBP, mmHg	135.78 ± 17.91	141.74 ± 18.75	134.97 ± 17.64	<0.001

Data are presented as mean ± standard deviation for continuous variables and *n* (%) for categorical variables. *t*-test was used for continuous variables and chi-square test for categorical variables. *p* < 0.05 was considered statistically significant. BMI: Body Mass Index; CVD: Cardiovascular Disease; CKM: cardiovascular-kidney-metabolic; eGFR: estimated glomerular filtration rate; HDL-C: High-Density Lipoprotein Cholesterol; LDL-C: Low-Density Lipoprotein Cholesterol; TG: Triglycerides; TC: Total Cholesterol; SUA: Serum Uric Acid; hs-CRP: High-Sensitivity C-Reactive Protein; DBP: Diastolic Blood Pressure; Sr: Serum creatinine; SBP: Systolic Blood Pressure; SII: Systemic Immune-Inflammation Index; SIRI: Systemic Immune-Inflammation Response Index.

With increasing quartiles of SII and SIRI at baseline, the proportions of individuals with CKM from stages 0 to 1 progressively decreased, while those from stages 2 to 3 increased (Supplementary Tables 3, 4).

### 3.2 Association of SII and SIRI with incidence of CVD

During a median follow-up of 13.0 years, a total CVD incidence occurred in 35,782 (11.86%) participants, including 18,398 CHD (6.10%), 8,578 CBVD (2.84%), 13,884 AF (4.60%), and 5,591 HF (1.85%), respectively.

The elevated SII was significantly associated with an increased risk of CVD incidence in individuals with CKM syndrome (Table 2). Compared to SII at quartile Q1, participants with SII at quartile Q4 had a high incidence of CVD (HR = 1.07, 95% CI: 1.04–1.10, *p* < 0.001). Each unit increase in SII was associated with an increased 7% risk in incidence of CVD (HR 1.07, 95% CI 1.05–1.10; *p* < 0.001).

**Table 2:** Adjusted HR (95% CI) for association between SII and incidence of CVD.

SII	Model 1	Model 2	Model 3
CVD			
Continuous	1.11 (1.08, 1.13)	1.10 (1.07, 1.12)	1.07 (1.05, 1.10)
Categories			
Q1	1	1	1
Q2	0.98 (0.96, 1.01)	0.99 (0.96, 1.02)	1.00 (0.96, 1.02)
Q3	1.01 (0.99, 1.05)	1.02 (0.99, 1.05)	1.01 (0.98, 1.04)
Q4	1.12 (1.09, 1.15)	1.10 (1.07, 1.13)	1.07 (1.04, 1.10)
p-trend	<0.001	<0.001	<0.001

Model 1 adjusted for age and sex; Model 2 additionally included ethnicity, socioeconomic status, education, alcohol use, smoking, sleep status, and physical activity; and Model 3 further adjusted for diabetes, hypertension, BMI, LDL-C, HDL-C, eGFR, and hs-CRP.

A positive association between SIRI and the risk of CVD incidence was observed (Table 3). Participants with SIRI at Q4 had a significantly higher risk of CVD incidence compared to those with SIRI at Q1 (HR = 1.19, 95% CI: 1.15–1.22; *p* < 0.001). Each unit increase in SIRI was associated with an increased 31% risk of CVD incidence (HR = 1.31, 95% CI: 1.26–1.37, *p* < 0.001).

**Table 3:** Adjusted HR (95% CI) for the association between SIRI and incidence of CVD.

SIRI	Model 1	Model 2	Model 3
CVD			
Continuous	1.43 (1.38, 1.49)	1.40 (1.35, 1.46)	1.31 (1.26, 1.37)
Categories			
Q1	1	1	1
Q2	1.07 (1.03, 1.10)	1.05 (1.02, 1.09)	1.05 (1.02, 1.09)
Q3	1.14 (1.11, 1.18)	1.13 (1.09, 1.16)	1.11 (1.07, 1.14)
Q4	1.26 (1.22, 1.30)	1.24 (1.19, 1.27)	1.19 (1.15, 1.22)
p-trend	<0.001	<0.001	<0.001

Model 1 adjusted for age and sex; Model 2 additionally included ethnicity, socioeconomic status, education, alcohol use, smoking, sleep status, and physical activity; and Model 3 further adjusted for diabetes, hypertension, BMI, LDL-C, HDL-C, eGFR, and hs-CRP.

### 3.3 RCS analyses

**Figure 2:**
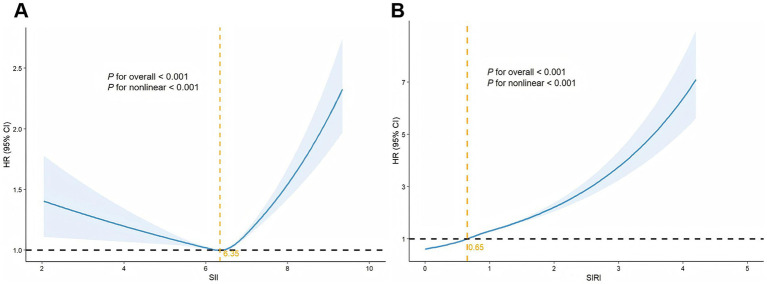
RCS analysis of SII and SIRI with CVD risk in individuals with CKM syndrome (**A** for SII, **B** for SIRI. Hazard ratios were adjusted for age, sex, race, Townsend deprivation index, education level, alcohol consumption, smoking status, sleep duration, physical activity, diabetes, hypertension, BMI, LDL-C, HDL-C, eGFR and hs-CRP).

RCS analyses revealed nonlinear associations between SII and SIRI with incidence of CVD (Figure 2). A U-shaped relationship was observed for SII. Below the inflection point of 6.35, SII was associated with a reduced risk of CVD incidence (HR = 0.92, 95% CI: 0.89–0.97; *p* < 0.001), whereas the risk increased significantly (HR = 1.13, 95% CI: 1.10–1.18; *p* < 0.001) above this threshold. SIRI exhibited a monotonically increasing association with incidence of CVD. The risk for CVD increased moderately following a 0.65 unit decrease in the slope (HR = 1.18, 95% CI: 1.14–1.22), and rose more steeply above this threshold (HR = 1.32, 95% CI: 1.27–1.40) (Table 4).

**Table 4:** Threshold analysis of the association of SII and SIRI with incidence of CVD in individuals with CKM syndrome.

CVD risk	Adjusted HR (95% CI)	*p*-value
SII		
Fitting using the Cox regression model	1.07 (1.05, 1.10)	<0.001
Fitting using the two-piecewise Cox regression model		
Inflection point	6.35	
SII < 6.35	0.92 (0.89, 0.97)	<0.001
SII ≥ 6.35	1.13 (1.10, 1.18)	<0.001
Log–likelihood ratio	<0.001	
SIRI		
Fitting using the Cox regression model	1.31 (1.26, 1.37)	<0.001
Fitting using the two-piecewise Cox regression model		
Inflection point	0.65	
SIRI < 0.65	1.18 (1.14, 1.22)	<0.001
SIRI ≥ 0.65	1.32 (1.27, 1.40)	<0.001
Log–likelihood ratio	<0.001	

Analyses were based on Model 3 with comprehensive adjustments, including age, sex, race, Townsend Deprivation Index, education level, alcohol consumption, smoking status, sleep duration, physical activity, diabetes, hypertension, BMI, LDL-C, HDL-C, and hs-CRP.

In stratified RCS analyses (Supplementary Figure 1), SII showed a consistent U-shaped association across sex, hypertension, and diabetes, with steeper gradients in men and those with comorbidities. SIRI displayed a monotonic increase in all subgroups, more pronounced among participants with hypertension and diabetes.

### 3.4 Subgroup analyses

After adjustment using Model 3, the association between SII and the incidence of CVD showed a graded increase across CKM stages 0 to 3, with corresponding HRs of 1.06 (95% CI: 1.01–1.12), 1.07 (1.04–1.13), 1.11 (1.08–1.15), and 1.14 (1.11–1.18), respectively. A similar dose–response pattern was observed for SIRI, with HRs of 1.22 (1.10–1.29), 1.28 (1.14–1.40), 1.39 (1.23–1.40), and 1.49 (1.33–1.66), respectively.

Additionally, we accounted for other variables, and no significant interactions were found for either SII or SIRI with age, sex, ethnicity, education, drinking, physical activity, BMI, or hypertension (all *p* for interaction > 0.05). In addition, the associations of SII and SIRI with incidence of CVD were significantly stronger in individuals with diabetes (*p* for interaction < 0.05) (Supplementary Figures 2, 3; Supplementary Tables 5, 6).

### 3.5 Mediation analyses

**Figure 3:**
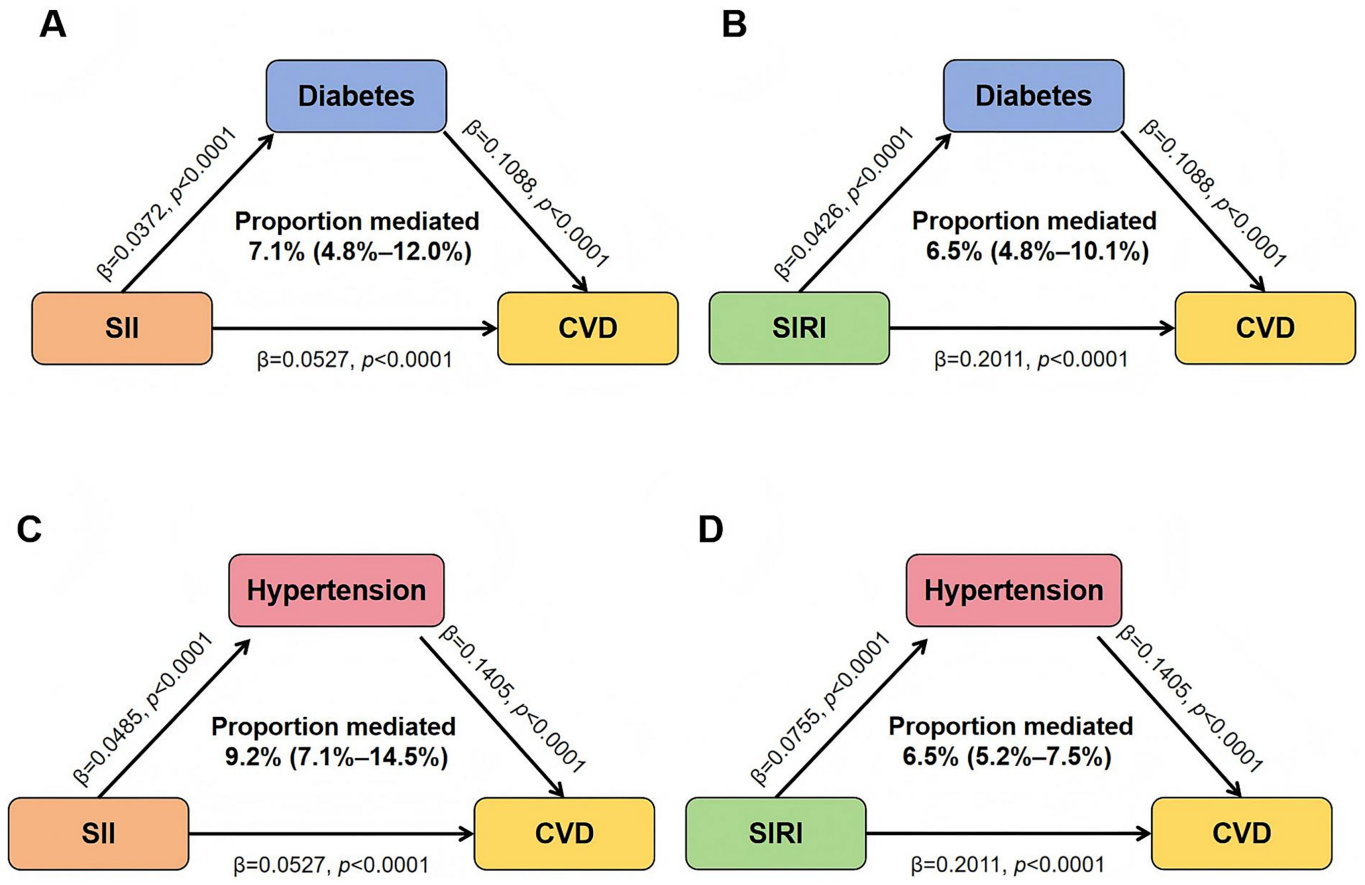
Mediation analyses of diabetes and hypertension in the associations between SII, SIRI, and CVD risk. **(A)** Diabetes mediating the association between SII and CVD. **(B)** Diabetes mediating the association between SIRI and CVD. **(C)** Hypertension mediating the association between SII and CVD. **(D)** Hypertension mediating the association between SIRI and CVD. All models were adjusted for age, sex, ethnicity, Townsend deprivation index, education level, alcohol consumption, smoking status, sleep duration, physical activity, BMI, LDL-C, HDL-C, eGFR, and hs-CRP.

To further explore potential pathways, we conducted mediation analyses (Figure 3). The results showed that both diabetes and hypertension significantly mediated the associations of SII and SIRI with incident CVD. Specifically, diabetes explained 7.1% (95% CI: 4.8–12.0%, *p* < 0.001) of the total effect for SII and 6.5% (95% CI: 4.8–10.1%, *p* < 0.001) for SIRI. Hypertension also demonstrated a modest mediating effect, accounting for approximately 9.2% (95% CI: 7.1–14.5%, *p* < 0.001) of SII and 6.5% (95% CI: 5.2–7.5%, *p* < 0.001) for SIRI. These findings suggest that diabetes and hypertension may serve as important pathways linking systemic inflammation to CVD risk in CKM syndrome. In contrast, obesity and hyperuricemia did not show significant mediation effects (all *p* > 0.05).

### 3.6 ROC analyses

**Figure 4:**
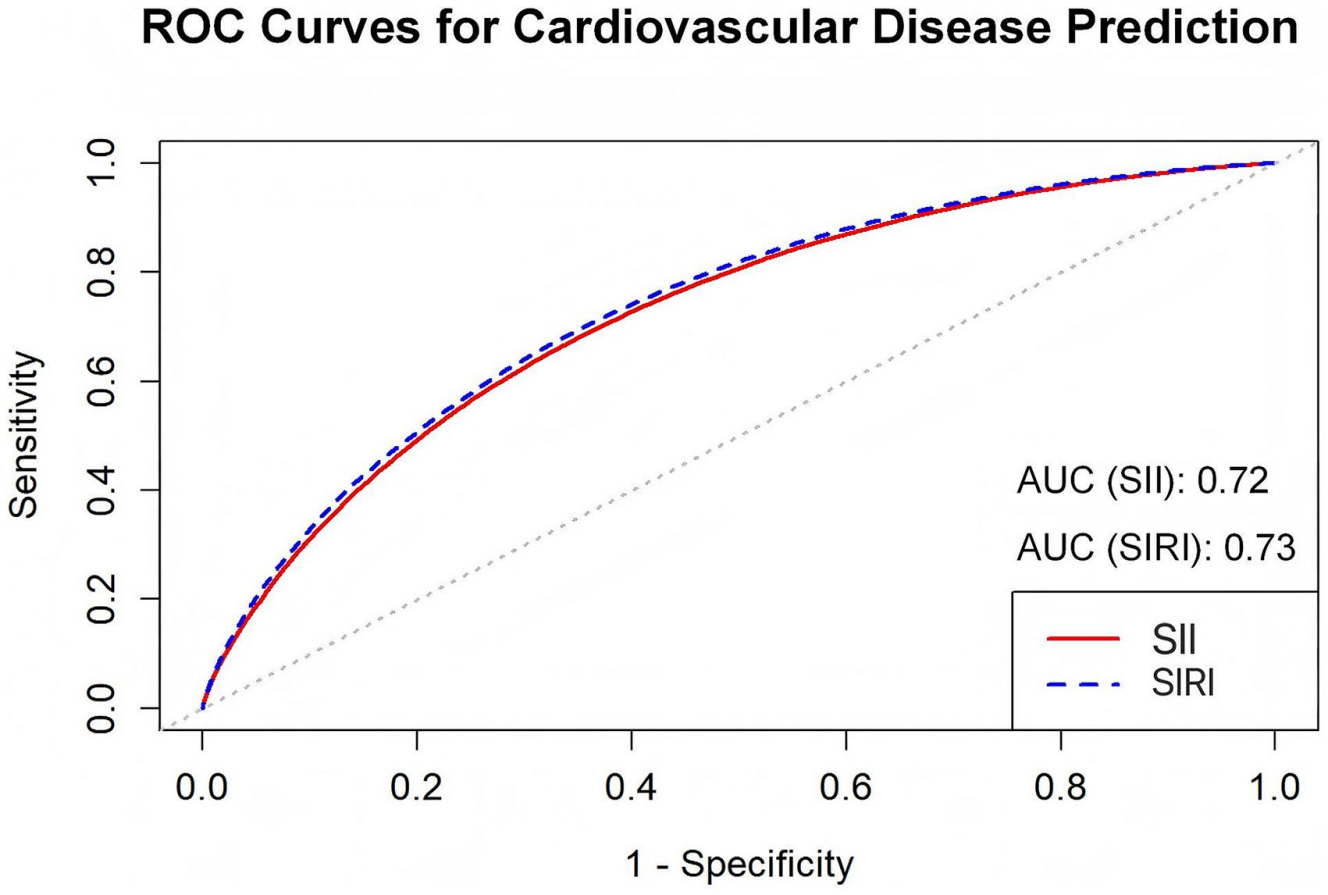
ROC curves for predicting incident CVD using SII and SIRI based on Model 3.

Both SII and SIRI demonstrated moderate discriminative ability for predicting incident CVD (Figure 4). The AUC for SII was 0.72 (95% CI: 0.70–0.74, *p* < 0.01), while that for SIRI was slightly higher at 0.73 (95% CI: 0.72–0.75, *p* < 0.01). Although the discriminative performance of SIRI was marginally better than SII, the difference between the two indices was small, indicating broadly comparable predictive ability.

### 3.7 Sensitivity analyses

Several sensitivity analyses were conducted. First, excluding 3,664 participants with <2 years of follow-up yielded similar positive associations of SII (HR = 1.08, 95% CI: 1.06–1.11) and SIRI (HR = 1.29, 95% CI: 1.24–1.35) with incidence of CVD (Supplementary Table 7). Second, PSM achieved adequate covariate balance (all standardized mean differences *p* < 0.1), and the associations remained consistent (SII: HR = 1.07, 95% CI: 1.05–1.10; SIRI: HR = 1.31, 95% CI: 1.26–1.36) (Supplementary Table 8). Third, using age as the timescale instead of follow-up duration, the associations remained consistent (SII: HR = 1.07, 95% CI: 1.05–1.09; SIRI: HR = 1.33, 95% CI: 1.28–1.39) (Supplementary Table 9). Fourth, after excluding CKM-defining variables from the adjustment set, the associations remained directionally consistent (SII: HR = 1.07, 95% CI: 1.05–1.09; SIRI: HR = 1.33, 95% CI: 1.28–1.39) (Supplementary Table 10).

## 4 Discussion

To our knowledge, this is the first study to systematically evaluate the association of SII and SIRI with incidence of CVD in a population with CKM syndrome, and the results indicated that the elevated SII and SIRI were associated with increased risk of CVD incidence. The strength of the association increased consecutively with CKM stages. Our results also showed that as CKM progresses, SII and SIRI, the key marks of the systemic immune-inflammatory, become more pronounced. ROC analyses suggested that both SII and SIRI demonstrated moderate discriminative ability for predicting incident CVD. Mediation analyses highlight the role of hypertension and diabetes in the association of SII and SIRI with CVD incidence.

The elevated SII and SIRI correspond to heightened neutrophil and monocyte activity, reduced lymphocyte counts, and platelet activation, which contribute to endothelial dysfunction, atherosclerotic plaque instability, and thrombosis (14–16). Neutrophils facilitate thrombosis and vascular injury via release of neutrophil extracellular traps (NETs) and reactive oxygen species (ROS) (17, 18). Monocytes promote atherosclerosis by transforming into macrophage-derived foam cells (19–21), whereas decreased lymphocyte counts imply diminished anti-inflammatory responses (22). Additionally, activated platelets amplify inflammatory cascades and enhance thrombotic risk (23, 24). SII and SIRI are composite indices derived from routine blood counts, reflecting systemic inflammation more comprehensively than single markers. Initially used in oncology, they have gained attention in cardiovascular research, especially in metabolically compromised populations like those with CKM syndrome. RCS analyses revealed distinct patterns for SII and SIRI. The U-shaped association of SII suggests that both very low and high platelet–neutrophil activity relative to lymphocytes may be harmful, reflecting impaired immunity at one extreme and excessive inflammation at the other. By contrast, SIRI showed a monotonic increase, consistent with progressive myeloid activation and lymphopenia aggravating atherosclerosis and thrombosis. These divergent patterns were robust across subgroups of sex, hypertension, and diabetes. Combined with ROC analyses, the findings further indicate that both indices have moderate and broadly comparable discriminative ability, with SIRI performing slightly better.

In CKM syndrome, high SII and SIRI may reflect the systemic inflammatory milieu driven by metabolic disturbances, adipose tissue dysfunction, and renal impairment (1, 25, 26). Dysfunctional visceral adiposity promotes macrophage polarization toward a pro-inflammatory M1 phenotype and activates inflammasome pathways, such as the TLR4/NF-κB pathway and the NLRP3 inflammasome, resulting in persistent elevations in circulating neutrophils and monocytes (27–30), the central components of SII and SIRI. Moreover, renal impairment intensifies systemic inflammation by reducing clearance of pro-inflammatory mediators and uremic toxins and further amplifying neutrophil-monocyte activation and oxidative stress (31–33). These biological processes may help explain our findings that the associations of SII and SIRI with CVD risk became progressively stronger across CKM stages. With advancing CKM severity, accumulating metabolic dysfunction and renal impairment amplify systemic inflammation. As a result, baseline SII and SIRI levels rise in parallel with CKM progression, reflecting the increasing immune-inflammatory burden.

Our mediation and subgroup analyses underscore the roles of both diabetes and hypertension as important clinical modifiers and mechanistic amplifiers of systemic inflammation. We observed that the associations between SII, SIRI, and incident CVD were consistently stronger among individuals with diabetes and those with hypertension, suggesting that poor glycemic control and elevated blood pressure may accelerate inflammatory activation. Moreover, mediation analyses demonstrated that both diabetes and hypertension partially mediated the relationships of SII and SIRI with CVD, supporting their role as causal pathways rather than mere correlates. Collectively, these findings highlight that the systemic inflammatory burden captured by SII and SIRI is magnified by metabolic and hemodynamic dysfunction, emphasizing the importance of integrated strategies that target systemic inflammation, glycemic dysregulation, and blood pressure control to mitigate cardiovascular risk in CKM populations.

The Kailuan cohort study suggested that the elevated SIRI was significantly associated with a higher incidence of ischemic stroke and MI (4). Another study, based on the NHANES database, reported that the elevation of SII was associated with CVD events (34). Our study extends the previous findings in several important ways: First, by targeting individuals with CKM syndrome, this population was characterized by heightened systemic inflammation and multisystem dysfunction, offering a novel insight beyond general population analyses. Second, the UKB data has an extensive covariate adjustment and long-term follow-up, which strengthened the reliability and generalizability of the findings.

The limitations in this study should be noticed. First, as an observational study, causal association can not be established. Second, SII and SIRI were only measured at baseline, preventing the assessment of inflammatory status across longitudinal changes. Third, while the UKB is a large and representative cohort, its predominantly UK-based and ethnically homogenous population may limit the generalizability of the findings to more diverse global populations.

## 5 Conclusion

In individuals with CKM syndrome, the elevated SII and SIRI were closely associated with the increased risks of CVD incidence. These findings offer a novel insight into systemic immune-inflammatory biomarkers for predicting incidence of CVD in the CKM population.

## Data Availability

The original contributions presented in the study are included in the article/Supplementary material, further inquiries can be directed to the corresponding authors.

## Supplementary material

SUPPLEMENTARY FIGURE 1 Stratified RCS analyses of the associations of SII and SIRI with incident CVD. **(A1–A3)** Associations between SII and CVD risk stratified by sex **(A1)**, hypertension status **(A2)**, and diabetes status **(A3)**. **(B1–B3)** Associations between SIRI and CVD risk stratified by sex **(B1)**, hypertension status **(B2)**, and diabetes status **(B3)**.

SUPPLEMENTARY FIGURE 2 Subgroup analyses of the association between SII and incident CVD.

SUPPLEMENTARY FIGURE 3 Subgroup analyses of the association between SIRI and incident CVD.

## Glossary

AF: Atrial fibrillation
AMI: Acute myocardial infarction
BMI: Body mass index
CHD: Coronary heart disease
CIHD: Chronic ischemic heart disease
CRP: C-reactive protein
CVD: Cardiovascular disease
CKM: cardiovascular-kidney-metabolic
DBP: Diastolic blood pressure
eGFR: estimated glomerular filtration rate
HDL-C: High-density lipoprotein cholesterol
HF: Heart failure
IL-6: Interleukin-6
LDL-C: Low-density lipoprotein cholesterol
NETs: Neutrophil extracellular traps
ROS: Reactive oxygen species
SBP: Systolic blood pressure
SII: Systemic immune-inflammation index
SIRI: Systemic inflammation response index
SUA: Serum uric acid
TC: Total cholesterol
TG: Triglycerides
UKB: UK Biobank
