# Supplementary figures and images for: Association of SII and SIRI with incidence of cardiovascular disease in cardiovascular-kidney-metabolic syndrome: a prospective cohort study
Source: Front Nutr. 2025 Nov 24;12:1661826. doi: 10.3389/fnut.2025.1661826 (PMC12683910; doi:10.3389/fnut.2025.1661826)

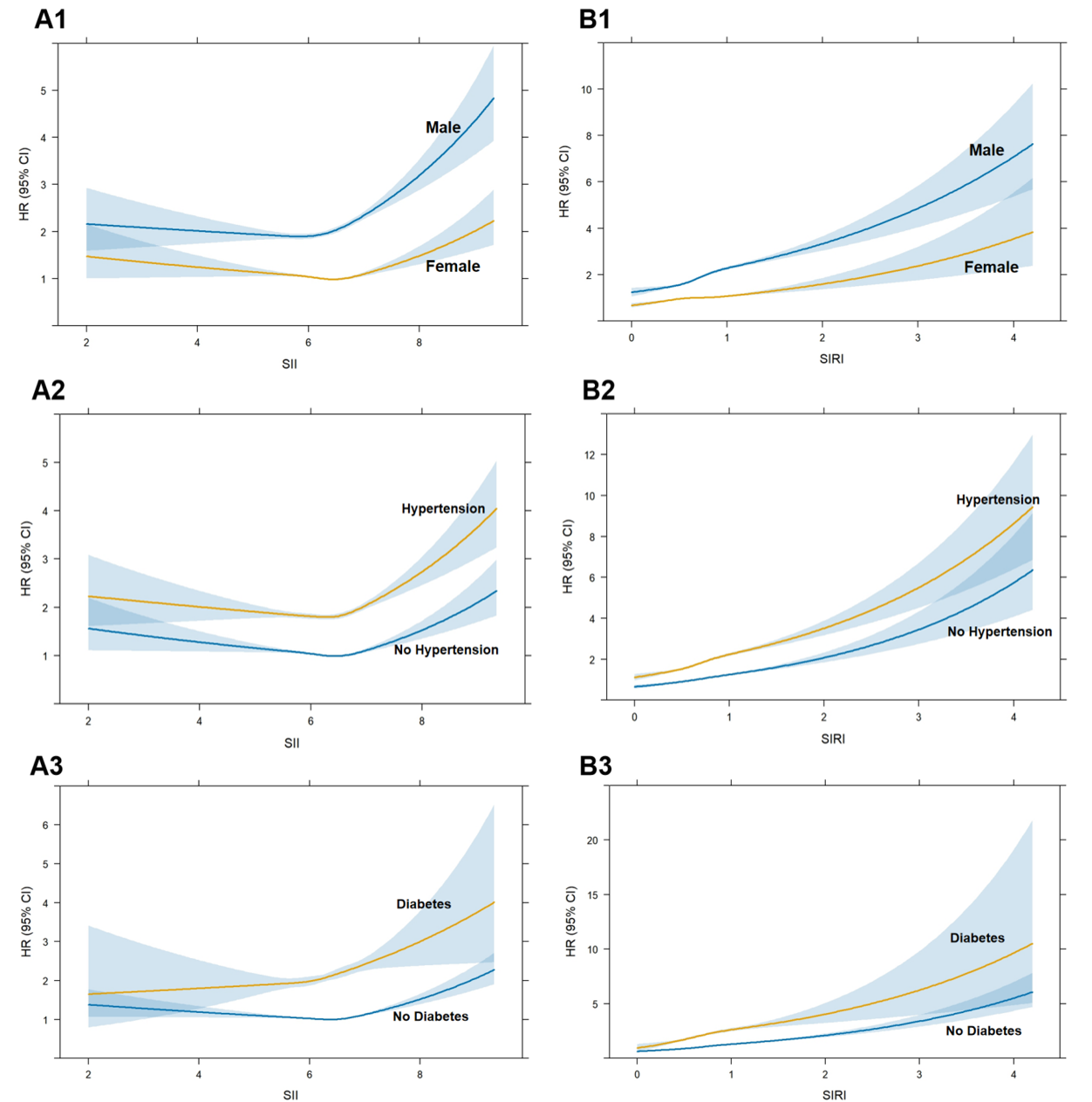

Supplement: SUPPLEMENTARY FIGURE 1 — Stratified RCS analyses of the associations of SII and SIRI with incident CVD. (A1–A3) Associations between SII and CVD risk stratified by sex (A1), hypertension status (A2), and diabetes status (A3). (B1–B3) Associations between SIRI and CVD risk stratified by sex (B1), hypertension status (B2), and diabetes status (B3). [file Image_1.tif]

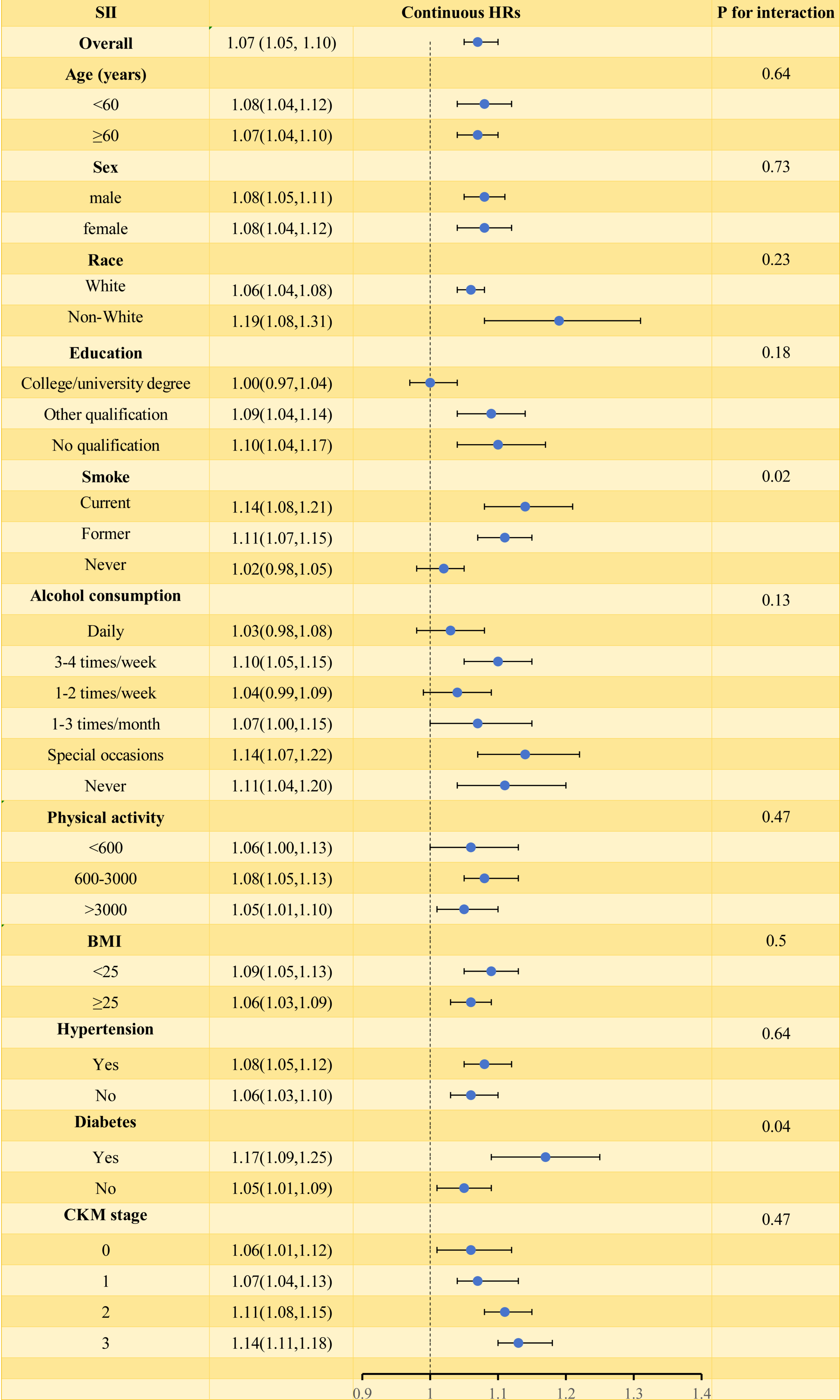

Supplement: SUPPLEMENTARY FIGURE 2 — Subgroup analyses of the association between SII and incident CVD. [file Image_2.tif]

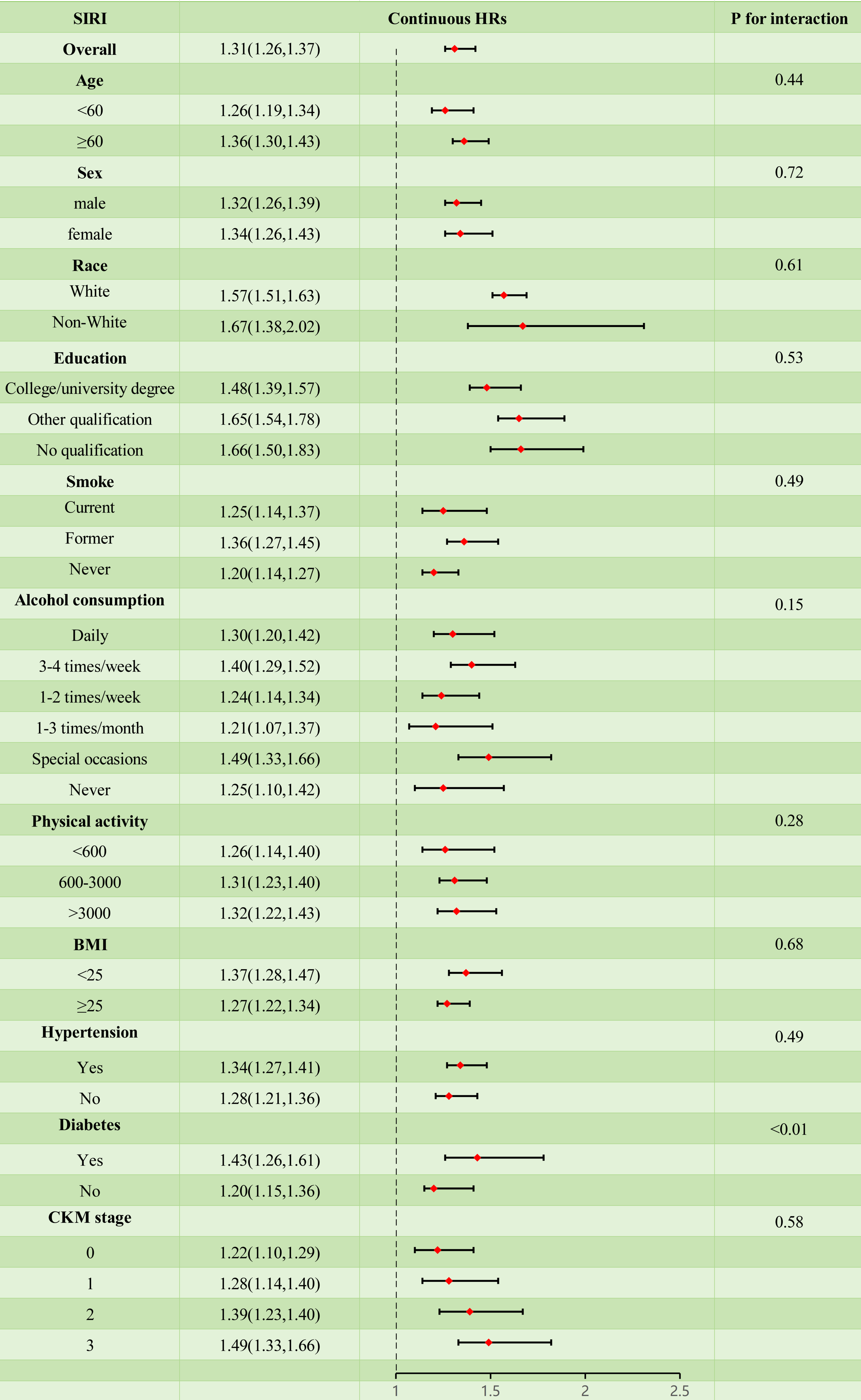

Supplement: SUPPLEMENTARY FIGURE 3 — Subgroup analyses of the association between SIRI and incident CVD. [file Image_3.tif]

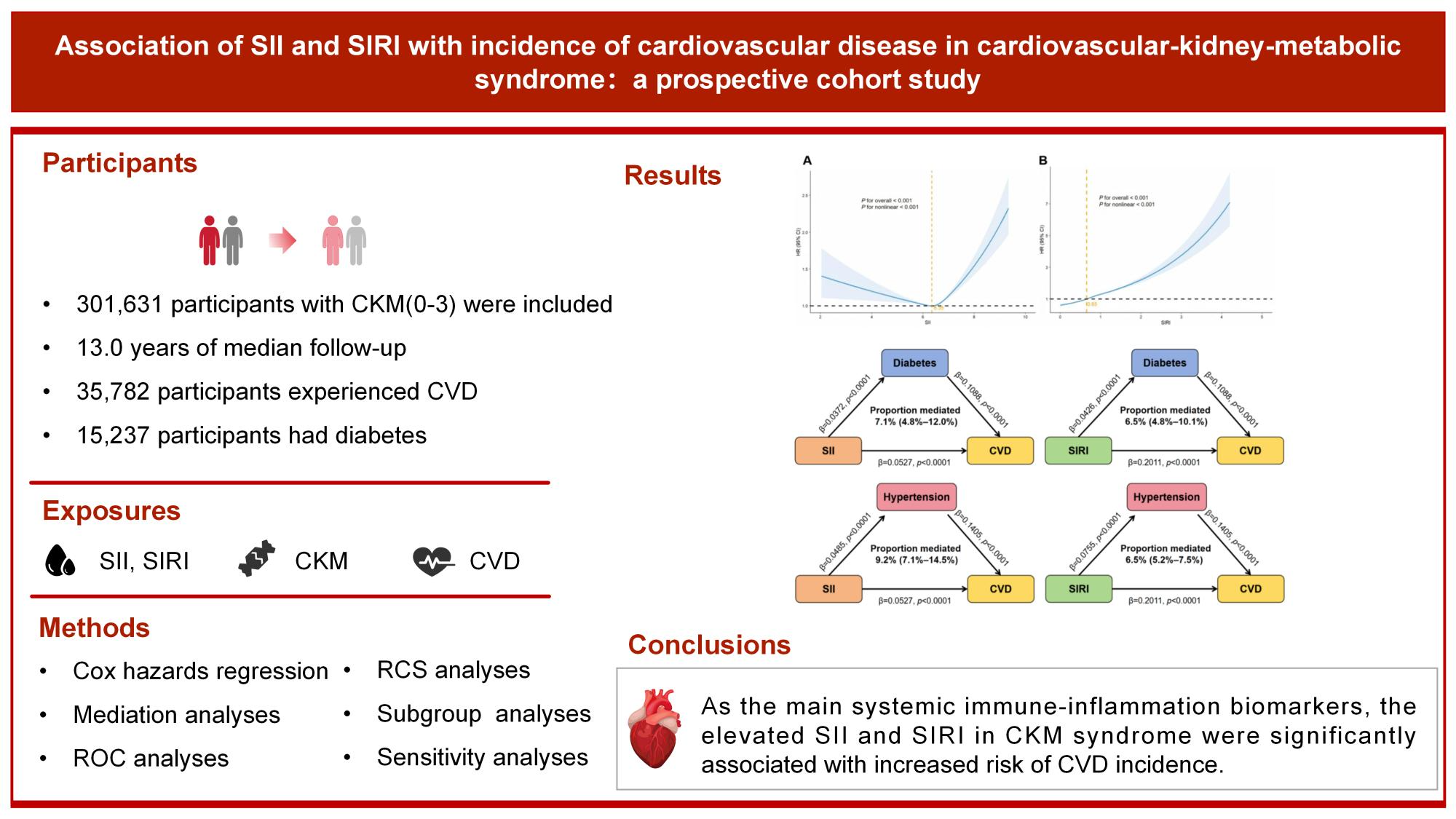

Supplement: Supplementary file 4 [file Image_4.tif]
