# Supplementary material for: Association of SII and SIRI with incidence of cardiovascular disease in cardiovascular-kidney-metabolic syndrome: a prospective cohort study
Source: Front Nutr. 2025 Nov 24;12:1661826. doi: 10.3389/fnut.2025.1661826 (PMC12683910; doi:10.3389/fnut.2025.1661826)
Supplement: Supplementary file 5 [file Table_1.doc]

**Supplementary Table 1. Specific staging criteria for CKM stages 0-3**

| CKM stages | Threshold for CKM conditions |
| --- | --- |
| Stages 0 | All criteria are met：   1. BMI< 23 kg/m2 2. Waist circumference <80/90 cm in female/male 3. Fasting blood glucose < 100 mg/dL and HbA1c < 5.7% and without self-reported diagnosis of diabetes, use of insulin, or oral hypoglycemic agents. 4. SBP <130 mm Hg and DBP <80 mm Hg without self-reported diagnosis of hypertension or use of antihypertensive medications. 5. HDL-C ≥50/40 mg/dL in female/male 6. TG < 150 mg/dL 7. eGFR ≥ 60 ml/min/1.73m2, and without self-reported diagnosis of CKD 8. No Subclinical CVD and clinical CVD |
| Stages 1 | Any of the three criteria is met:   1. Overweight/obesity 2. Abdominal obesity 3. Prediabetes   All criteria are met：  （1）SBP <130 mmHg and DBP <80 mmHg without self-reported diagnosis of hypertension or use of antihypertensive medications.  （2）HDL-C ≥ 50/40 mg/dL in female/male  （3）TG < 150 mg/dL  （4）eGFR ≥ 60 ml/min/1.73m2, and without self-reported diagnosis of CKD  （5）No Subclinical CVD and clinical CVD |
| Stages 2 | Any of the five criteria is met:   1. Hypertriglyceridemia 2. Hypertension 3. Diabetes 4. metabolic syndrome 5. eGFR：30-60 ml/min/1.73m2, and/or with self-reported diagnosis of CKD   All criteria are met：   1. No Subclinical CVD and clinical CVD |
| Stages 3 | Any of the two criteria is met:   1. eGFR<30 ml/min/1.73m2 2. Subclinical CVD   Any of the eight criteria is met:   1. Overweight/obesity 2. Abdominal obesity 3. Prediabetes 4. Hypertriglyceridemia 5. Hypertension 6. diabetes 7. metabolic syndrome 8. eGFR：30-60 ml/min/1.73m2, and/or with self-reported diagnosis of CKD   The criterion is met：   1. No clinical CVD |

Abbreviations: BMI - Body Mass Index; CVD - Cardiovascular Disease; DBP - Diastolic Blood Pressure; eGFR - Estimated Glomerular Filtration Rate; HbA1c - Hemoglobin A1c; HDL-C - High-Density Lipoprotein Cholesterol; SBP - Systolic Blood Pressure; TG - Triglycerides.

**Supplementary Table 2. Collinearity statistics of covariates in the Cox regression model**

| **Variables** | **VIF** |
| --- | --- |
| Age | 2.8 |
| Sex | 2.1 |
| Ethnicity | 1.2 |
| Education | 1.0 |
| Townsend deprivation index | 1.1 |
| Smoking | 2.5 |
| Alcohol use | 1.2 |
| Sleep status | 0.9 |
| Physical activity | 1.1 |
| Diabetes | 1.8 |
| Hypertension | 1.8 |
| BMI | 1.9 |
| HDL-C | 1.5 |
| LDL-C | 1.5 |
| eGFR | 2.2 |
| hs-CRP | 3.4 |

Abbreviations: BMI – Body Mass Index; eGFR – Estimated Glomerular Filtration Rate; HDL-C: High-Density Lipoprotein Cholesterol; LDL-C: Low-Density Lipoprotein Cholesterol; hs-CRP: High-Sensitivity C-Reactive Protein.

**Supplementary Table 3. Baseline characteristics of study participants stratified by SII quartiles**

| **Characteristic** | **N = 301,631** | **Q1,N = 75,470 (<5.97)** | **Q2,N = 75,516 (5.97-6.26)** | **Q3,N = 75,652 (6.26-6.56)** | **Q4,N = 74,993 (>6.56)** | **p-value** |
| --- | --- | --- | --- | --- | --- | --- |
| **Age (years)** | 54.86±8.12 | 55.21±7.98 | 55.08±8.02 | 54.76±8.14 | 54.38±8.32 | <0.001 |
| **Sex, n (%)** |  |  |  |  |  | <0.001 |
| Female | 170,708(56.6%) | 39,844(52.8%) | 42,530(56.3%) | 43,866(58.0%) | 44,468(59.3%) |  |
| Male | 130,923(43.4%) | 35,626(47.2%) | 32,986(43.7%) | 31,786(42.0%) | 30,525(40.7%) |  |
| **Race, n (%)** |  |  |  |  |  | <0.001 |
| White | 284,956(94.5%) | 68,542(90.8%) | 71,643(94.9%) | 72,490(95.8%) | 72,281(96.4%) |  |
| Not White | 16,675(5.5%) | 6,928(9.2%) | 3,873(5.1%) | 3,162(4.2%) | 2,712(3.6%) |  |
| **Education, n (%)** |  |  |  |  |  | <0.001 |
| College/university degree | 142,851(47.4%) | 37,197(49.3%) | 36,319(48.1%) | 35,489(46.9%) | 33,846(45.1%) |  |
| Other qualification | 84,967(28.2%) | 20,124(26.7%) | 20,931(27.7%) | 21,765(28.8%) | 22,147(29.5%) |  |
| No qualification | 32,785(10.9%) | 8,453(11.2%) | 8,214(10.9%) | 8,079(10.7%) | 8,039(10.7%) |  |
| Unknown | 41,028(13.6%) | 9,696(12.8%) | 10,052(13.3%) | 10,319(13.6%) | 10,961(14.6%) |  |
| **Alcohol consumption, n (%)** |  |  |  |  |  | <0.001 |
| Daily | 60,266(20.0%) | 15,294(20.3%) | 15,529(20.6%) | 14,931(19.7%) | 14,512(19.4%) |  |
| 3-4 times/week | 72,543(24.1%) | 18,604(24.7%) | 18,449(24.4%) | 18,305(24.2%) | 17,185(22.9%) |  |
| 1-2 times/week | 80,329(26.6%) | 19,738(26.2%) | 20,060(26.6%) | 20,349(26.9%) | 20,182(26.9%) |  |
| 1-3 times/month | 34,371(11.4%) | 8,076(10.7%) | 8,473(11.2%) | 8,870(11.7%) | 8,952(11.9%) |  |
| Special occasions | 32,387(10.7%) | 8,018(10.6%) | 7,833(10.4%) | 8,025(10.6%) | 8,511(11.3%) |  |
| Never | 21,735(7.2%) | 5,740(7.6%) | 5,172(6.8%) | 5,172(6.8%) | 5,651(7.5%) |  |
| **Physical activity (MET-minutes/week)** |  |  |  |  |  | <0.001 |
| <600 | 41,097(13.6%) | 9,637(12.8%) | 10,147(13.4%) | 10,515(13.9%) | 10,798(14.4%) |  |
| 600-3000 | 185,181(61.4%) | 46,081(61.1%) | 46,371(61.4%) | 46,493(61.5%) | 46,236(61.7%) |  |
| >3000 | 75,353(25.0%) | 19,75226.2%) | 18,998(25.2%) | 18,644(24.6%) | 17,959(23.9%) |  |
| **Smoke, n (%)** |  |  |  |  |  | <0.001 |
| Never | 173,449(57.5%) | 43,397(57.5%) | 43,365(57.4%) | 43,658(57.7%) | 43,029(57.4%) |  |
| Former | 95,625(31.7%) | 24,604(32.6%) | 24,507(32.5%) | 23,722(31.4%) | 22,792(30.4%) |  |
| Current | 32,557(10.8%) | 7,469(9.9%) | 7,644(10.1%) | 8,272(10.9%) | 9,172(12.2%) |  |
| **CHD, n (%)** |  |  |  |  |  | <0.001 |
| **Yes** | 265,849(88.1%) | 66,523(88.1%) | 66,774(88.4%) | 66,820(88.3%) | 65,732(87.7%) |  |
| **No** | 35,782(11.9%) | 8,947(11.9%) | 8,742(11.6%) | 8,832(11.7%) | 9,261(12.3%) |  |
| **CKM stages, n (%)** |  |  |  |  |  | <0.001 |
| 0 | 42,530 (14.1%) | 11,996 (15.9%) | 11,685 (15.5%) | 10,868 (14.4%) | 7,981 (10.6%) |  |
| 1 | 58,516 (19.4%) | 18,702 (24.8%) | 15,108 (20.0%) | 13,891 (18.4%) | 10,815 (14.4%) |  |
| 2 | 171,024 (56.7%) | 41,138 (54.5%) | 41,774 (55.3%) | 42,165 (55.7%) | 45,947 (61.3%) |  |
| 3 | 29,561 (9.8%) | 3,634 (4.8%) | 6,949 (9.2%) | 8,728 (11.5%) | 10,250 (13.7%) |  |
| **Diabetes, n (%)** | 15,237(5.1%) | 4,184(5.5%) | 3,630(4.8%) | 3,619(4.8%) | 3,804(5.1%) | <0.001 |
| **Hypertension, n (%)** | 117,941(39.1%) | 28,862(38.2%) | 29,219(38.7%) | 29,460(38.9%) | 30,400(40.5%) | <0.001 |
| **Townsend Deprivation Index** | -1.43±3.01 | -1.39±3.05 | -1.51±2.97 | -1.48±2.97 | -1.35±3.06 | <0.001 |
| **Sleep duration (hours/day)** | 7.15±1.03 | 7.13±1.01 | 7.15±1.01 | 7.15±1.02 | 7.15±1.06 | <0.001 |
| **BMI, kg/m2** | 26.61±4.35 | 26.59±4.16 | 26.59±4.21 | 26.69±4.36 | 26.59±4.64 | <0.001 |
| **Lymphocyte, ×10**⁹ **cells/L** | 1.94±0.72 | 2.24±1.03 | 2.01±0.55 | 1.86±0.51 | 1.63±0.50 | <0.001 |
| **Monocyte, ×10**⁹ **cells/L** | 0.46±0.21 | 0.46±0.26 | 0.45±0.15 | 0.46±0.20 | 0.47±0.18 | <0.001 |
| **Neutrophil, ×10**⁹ **cells/L** | 4.12±1.37 | 3.11±0.88 | 3.77±0.91 | 4.30±1.03 | 5.31±1.50 | <0.001 |
| **Platelet, ×10**⁹ **cells/L** | 253.88±58.64 | 215.70±46.55 | 243.73±44.81 | 262.66±48.55 | 293.68±63.36 | <0.001 |
| **SIRI** | 1.06±0.94 | 0.65±0.30 | 0.87±0.34 | 1.09±0.47 | 1.64±1.60 | <0.001 |
| **HDL-C, mmol/L** | 1.48±0.36 | 1.48±0.36 | 1.48±0.36 | 1.48±0.35 | 1.47±0.35 | <0.001 |
| **LDL-C, mmol/L** | 3.65±0.81 | 3.65±0.81 | 3.68±0.81 | 3.66±0.81 | 3.62±0.82 | <0.001 |
| **TG, mmol/L** | 1.66±0.97 | 1.64±0.99 | 1.67±1.00 | 1.68±0.98 | 1.64±0.93 | <0.001 |
| **TC, mmol/L** | 5.83±1.06 | 5.82±1.06 | 5.86±1.06 | 5.84±1.05 | 5.78±1.06 | <0.001 |
| **SUA, µmol/L** | 297.18±74.22 | 300.13±74.79 | 297.63±74.06 | 296.35±73.66 | 294.61±74.26 | <0.001 |
| **Sr, µmol/L** | 70.91±13.73 | 71.81±13.52 | 71.02±13.35 | 70.68±13.44 | 70.14±14.52 | <0.001 |
| **Urea, mmol/L** | 5.26±1.20 | 5.30±1.18 | 5.30±1.19 | 5.25±1.20 | 5.17±1.24 | <0.001 |
| **hs-CRP, mg/L** | 2.25±3.88 | 1.66±2.50 | 1.88±2.88 | 2.22±3.48 | 3.24±5.68 | <0.001 |
| **Fasting Glucose, mmol/L** | 4.99±0.96 | 4.98±0.93 | 4.98±0.92 | 4.98±0.94 | 5.02±1.04 | <0.001 |
| **HbA1c, %** | 35.14±5.39 | 35.30±5.65 | 35.13±5.23 | 35.07±5.24 | 35.08±5.42 | <0.001 |
| **eGFR, mL/min/1.73m2** | 94.18 ± 12.25 | 94.96 ± 14.13 | 94.34 ± 14.11 | 93.84 ± 13.12 | 93.87 ± 13.57 | <0.001 |
| **DBP, mmHg** | 80.68±9.89 | 80.42±9.85 | 80.57±9.85 | 80.70±9.84 | 81.05±10.01 | <0.001 |
| **SBP, mmHg** | 135.78±17.91 | 135.34±17.61 | 135.55±17.77 | 135.79±17.94 | 136.43±18.28 | <0.001 |

Data are presented as mean ± standard deviation for continuous variables and n (%) for categorical variables. t-test for continuous variables and chi-square test for categorical variables. p < 0.05 was considered statistically significant.

BMI: Body Mass Index; CVD: Cardiovascular Disease; CKM: cardiovascular-kidney-metabolic; eGFR: estimated glomerular filtration rate; HDL-C: High-Density Lipoprotein Cholesterol; LDL-C: Low-Density Lipoprotein Cholesterol; HbA1c: Glycated Hemoglobin; TG: Triglycerides; TC: Total Cholesterol; SUA: Serum Uric Acid; hs-CRP: High-Sensitivity C-Reactive Protein; DBP: Diastolic Blood Pressure; Sr: Serum creatinine; SBP: Systolic Blood Pressure; SIRI: Systemic Immune-Inflammation Response Index.

**Supplementary Table 4. Baseline characteristics of study participants stratified by SIRI quartiles**

| **Characteristic** | **N = 301,631** | **Q1,N = 75,281 (<0.50)** | **Q2,N = 75,615 (0.50-0.65)** | **Q3,N = 75,552 (0.65-0.83)** | **Q4,N = 75,183 (>0.83)** | **p-value** |
| --- | --- | --- | --- | --- | --- | --- |
| **Age (years)** | 54.86±8.12 | 54.58±7.82 | 54.81±8.05 | 54.85±8.21 | 55.20±8.39 | <0.001 |
| **Sex, n (%)** |  |  |  |  |  | <0.001 |
| Female | 170,708(56.6%) | 51,569(68.5%) | 45,888(60.7%) | 40,633(53.8%) | 32,618(43.4%) |  |
| Male | 130,923(43.4%) | 23,712(31.5%) | 29,727(39.3%) | 34,919(46.2%) | 42,565(56.6%) |  |
| **Race, n (%)** |  |  |  |  |  | <0.001 |
| White | 284,956(94.5%) | 68,078(90.4%) | 71,656(94.8%) | 72,456(95.9%) | 72,766(96.8%) |  |
| Not White | 16,675(5.5%) | 7,203(9.6%) | 3,959(5.2%) | 3,096(4.1%) | 2,417(3.2%) |  |
| **Education, n (%)** |  |  |  |  |  | <0.001 |
| College/university degree | 142,851(47.4%) | 37,567(49.9%) | 36,590(48.4%) | 35,454(46.9%) | 33,240(44.2%) |  |
| Other qualification | 84,967(28.2%) | 20,531(27.3%) | 21,294(28.2%) | 21,433(28.4%) | 21,709(28.9%) |  |
| No qualification | 32,785(10.9%) | 7,854(10.4%) | 7,953(10.5%) | 8,350(11.1%) | 8,628(11.5%) |  |
| Unknown | 41,028(13.6%) | 9,329(12.4%) | 9,778(12.9%) | 10,315(13.7%) | 11,606(15.4%) |  |
| **Alcohol consumption, n (%)** |  |  |  |  |  | <0.001 |
| Daily | 60,266(20.0%) | 14,037(18.6%) | 14,838(19.6%) | 15,226(20.2%) | 16,165(21.5%) |  |
| 3-4 times/week | 72,543(24.1%) | 17,984(23.9%) | 18,527(24.5%) | 18,347(24.3%) | 17,685(23.5%) |  |
| 1-2 times/week | 80,329(26.6%) | 19,859(26.4%) | 20,158(26.7%) | 20,226(26.8%) | 20,086(26.7%) |  |
| 1-3 times/month | 34,371(11.4%) | 8,523(11.3%) | 8,676(11.5%) | 8,705(11.5%) | 8,467(11.3%) |  |
| Special occasions | 32,387(10.7%) | 8,912(11.8%) | 7,956(10.5%) | 7,836(10.4%) | 7,683(10.2%) |  |
| Never | 21,735(7.2%) | 5,966(7.9%) | 5,460(7.2%) | 5,212(6.9%) | 5,097(6.8%) |  |
| **Physical activity (MET-minutes/week)** |  |  |  |  |  | <0.001 |
| <600 | 41,097(13.6%) | 9,393(12.5%) | 10,185(13.5%) | 10,497(13.9%) | 11,022(14.7%) |  |
| 600-3000 | 185,181(61.4%) | 46,634(61.9%) | 46,496(61.5%) | 46,382(61.4%) | 45,669(60.7%) |  |
| >3000 | 75,353(25.0%) | 19,254(25.6%) | 18,934(25.0%) | 18,673(24.7%) | 18,492(24.6%) |  |
| **Smoke, n (%)** |  |  |  |  |  | <0.001 |
| Never | 173,449(57.6%) | 45,477(60.4%) | 44,196(58.4%) | 43,245(57.2%) | 40,531(53.9%) |  |
| Former | 95,625(31.7%) | 23,719(31.5%) | 24,243(32.1%) | 23,972(31.7%) | 23,691(31.5%) |  |
| Current | 32,557(10.8%) | 6,085(8.1%) | 7,176(9.5%) | 8,335(11.0%) | 10,961(14.6%) |  |
| **CVD, n (%)** |  |  |  |  |  | <0.001 |
| **Yes** | 265,849(88.1%) | 68,143(90.5%) | 67,368(89.1%) | 66,242(87.7%) | 64,096(85.3%) |  |
| **No** | 35,782(11.9%) | 7,138(9.5%) | 8,247(10.9%) | 9,310(12.3%) | 11,087(14.7%) |  |
| **CKM stages, n (%)** |  |  |  |  |  | <0.001 |
| 0 | 42,530 (14.1%) | 12,867 (17.1%) | 11,823 (15.6%) | 9,884 (13.1%) | 7,956 (10.6%) |  |
| 1 | 58,516 (19.4%) | 17,112 (22.7%) | 15,808 (20.9%) | 14,577 (19.3%) | 11,019 (14.7%) |  |
| 2 | 171,024 (56.7%) | 40,203 (53.4%) | 41,394 (54.7%) | 43,660 (57.8%) | 45,767 (60.9%) |  |
| 3 | 29,561 (9.8%) | 5,099 (6.8%) | 6,590 (8.7%) | 7,431 (9.8%) | 10,441(13.9%) |  |
| **Diabetes, n (%)** | 15,237(5.1%) | 3,466(4.6%) | 3,430(4.5%) | 3,796(5.0%) | 4,545(6.0%) | <0.001 |
| **Hypertension, n (%)** | 117,941(39.1%) | 26,102(34.7%) | 28,640(37.9%) | 30,314(40.1%) | 32,885(43.7%) | <0.001 |
| **Townsend Deprivation Index** | -1.43±3.01 | -1.38±3.07 | -1.53±2.95 | -1.47±3.00 | -1.34±3.04 | <0.001 |
| **Sleep duration (hours/day)** | 7.15±1.03 | 7.13±1.02 | 7.15±1.01 | 7.15±1.02 | 7.15±1.05 | <0.001 |
| **BMI, kg/m2** | 26.61±4.35 | 26.18±4.14 | 26.52±4.22 | 26.79±4.38 | 26.97±4.60 | <0.001 |
| **Lymphocyte, ×10**⁹ **cells/L** | 1.94±0.72 | 2.09±0.98 | 1.99±0.58 | 1.92±0.56 | 1.75±0.63 | <0.001 |
| **Monocyte, ×10**⁹ **cells/L** | 0.46±0.21 | 0.33±0.15 | 0.42±0.13 | 0.49±0.14 | 0.60±0.27 | <0.001 |
| **Neutrophil, ×10**⁹ **cells/L** | 4.12±1.37 | 3.10±0.94 | 3.74±0.88 | 4.30±0.99 | 5.34±1.47 | <0.001 |
| **Platelet, ×10**⁹ **cells/L** | 253.88±58.64 | 246.50±55.70 | 252.40±55.99 | 255.88±57.99 | 260.76±63.63 | <0.001 |
| **SII** | 586.73±335.95 | 390.30±179.22 | 495.95±176.99 | 602.15±213.87 | 859.22±470.92 | <0.001 |
| **HDL-C, mmol/L** | 1.48±0.36 | 1.54±0.36 | 1.49±0.36 | 1.46±0.35 | 1.42±0.35 | <0.001 |
| **LDL-C, mmol/L** | 3.65±0.81 | 3.70±0.82 | 3.68±0.81 | 3.65±0.81 | 3.58±0.81 | <0.001 |
| **TG, mmol/L** | 1.66±0.97 | 1.52±0.90 | 1.64±0.97 | 1.71±1.00 | 1.76±1.01 | <0.001 |
| **TC, mmol/L** | 5.83±1.06 | 5.91±1.07 | 5.87±1.06 | 5.81±1.05 | 5.70±1.05 | <0.001 |
| **SUA, µmol/L** | 297.18±74.22 | 284.20±70.89 | 293.23±72.71 | 300.56±74.26 | 310.78±76.33 | <0.001 |
| **Sr, µmol/L** | 70.91±13.73 | 68.84±12.74 | 70.17±13.17 | 71.44±13.46 | 73.20±15.06 | <0.001 |
| **Urea, mmol/L** | 5.26±1.20 | 5.16±1.16 | 5.25±1.18 | 5.29±1.21 | 5.32±1.26 | <0.001 |
| **hs-CRP, mg/L** | 2.25±3.88 | 1.63±2.52 | 1.88±2.77 | 2.21±3.40 | 3.27±5.76 | <0.001 |
| **Fasting Glucose, mmol/L** | 4.99±0.96 | 4.98±0.89 | 4.99±0.95 | 4.98±0.94 | 5.00±1.05 | <0.001 |
| **HbA1c, %** | 35.14±5.39 | 35.06±5.25 | 35.06±5.32 | 35.11±5.36 | 35.35±5.62 | <0.001 |
| **eGFR, mL/min/1.73m2** | 94.18 ± 12.25 | 94.97 ± 14.25 | 94.46 ± 14.19 | 93.41 ± 13.26 | 93.33 ± 13.22 | <0.001 |
| **Diastolic blood pressure, mmHg** | 80.68±9.89 | 79.81±9.73 | 80.38±9.75 | 80.93±9.87 | 81.62±10.11 | <0.001 |
| **Systolic blood pressure, mmHg** | 135.78±17.91 | 133.77±17.55 | 135.20±17.69 | 136.26±17.87 | 137.88±18.25 | <0.001 |

Data are presented as mean ± standard deviation for continuous variables and n (%) for categorical variables. t-test for continuous variables and chi-square test for categorical variables. p < 0.05 was considered statistically significant.

BMI: Body Mass Index; CVD: Cardiovascular Disease; CKM: cardiovascular-kidney-metabolic; eGFR: estimated glomerular filtration rate; HDL-C: High-Density Lipoprotein Cholesterol; LDL-C: Low-Density Lipoprotein Cholesterol; HbA1c: Glycated Hemoglobin; TG: Triglycerides; TC: Total Cholesterol; SUA: Serum Uric Acid; hs-CRP: High-Sensitivity C-Reactive Protein; DBP: Diastolic Blood Pressure; Sr: Serum creatinine; SBP: Systolic Blood Pressure; SII: Systemic Immune-Inflammation Index.

**Supplementary Table 5. Subgroup analyses of the association of SII with incidence of CVD in individuals with CKM syndrome**

| **SII** | **Continuous** | **Q1** | **Q2** | **Q3** | **Q4** | **P for trend** | **P for interaction** |
| --- | --- | --- | --- | --- | --- | --- | --- |
| **Age (years)** |  |  |  |  |  |  | 0.64 |
| <60 | 1.08(1.04,1.12) | 1 | 1.01(0.96,1.06) | 1.03(0.98,1.07) | 1.08(1.03,1.13) | <0.001 |  |
| ≥60 | 1.07(1.04,1.10) | 1 | 0.98(0.94,1.02) | 1.00(0.96,1.04) | 1.07(1.03,1.11) | <0.001 |  |
| **Sex** |  |  |  |  |  |  | 0.73 |
| male | 1.08(1.05,1.11) | 1 | 0.99(0.96,1.03) | 1.03(0.99,1.07) | 1.08(1.04,1.12) | <0.001 |  |
| female | 1.08(1.04,1.12) | 1 | 0.99(0.95,1.04) | 0.99(0.95,1.04) | 1.08(1.04,1.14) | <0.001 |  |
| **Race** |  |  |  |  |  |  | 0.23 |
| White | 1.06(1.04,1.08) | 1 | 0.97(0.94,1.00) | 0.98(0.95,1.01) | 1.05(1.02,1.08) | <0.001 |  |
| Non-White | 1.19(1.08,1.31) | 1 | 1.03(0.91,1.18) | 1.06(0.92,1.22) | 1.19(1.03,1.38) | <0.001 |  |
| **Education** |  |  |  |  |  |  | 0.18 |
| College/university degree | 1.00(0.97,1.04) | 1 | 0.99(0.94,1.04) | 0.97(0.93,1.02) | 1.00(0.96,1.05) | 0.10 |  |
| Other qualification | 1.09(1.04,1.14) | 1 | 0.95(0.90,1.01) | 0.97(0.92,1.03) | 1.08(1.02,1.14) | <0.001 |  |
| No qualification | 1.10(1.04,1.17) | 1 | 0.95(0.87,1.02) | 1.04(0.96,1.12) | 1.06(0.98,1.15) | <0.001 |  |
| **Smoke** |  |  |  |  |  |  | 0.02 |
| Current | 1.14(1.08,1.21) | 1 | 1.05(0.97,1.14) | 1.09(1.01,1.18) | 1.17(1.08,1.26) | <0.001 |  |
| Former | 1.11(1.07,1.15) | 1 | 1.02(0.97,1.07) | 1.02(0.97,1.07) | 1.11(1.05,1.16) | <0.001 |  |
| Never | 1.02(0.98,1.05) | 1 | 0.95(0.91,0.99) | 0.97(0.93,1.01) | 1.00(0.96,1.05) | 0.08 |  |
| **Alcohol consumption** |  |  |  |  |  |  | 0.13 |
| Daily | 1.03(0.98,1.08) | 1 | 0.97(0.91,1.03) | 1.00(0.94,1.07) | 1.02(0.96,1.09) | 0.08 |  |
| 3-4 times/week | 1.10(1.05,1.15) | 1 | 1.01(0.95,1.07) | 0.99(0.93,1.05) | 1.10(1.03,1.17) | <0.001 |  |
| 1-2 times/week | 1.04(0.99,1.09) | 1 | 0.99(0.93,1.05) | 1.02(0.97,1.09) | 1.03(0.97,1.09) | 0.06 |  |
| 1-3 times/month | 1.07(1.00,1.15) | 1 | 0.95(0.86,1.04) | 1.03(0.94,1.13) | 1.06(0.96,1.16) | 0.05 |  |
| Special occasions | 1.14(1.07,1.22) | 1 | 1.04(0.95,1.14) | 0.98(0.90,1.07) | 1.16(1.06,1.26) | <0.001 |  |
| Never | 1.11(1.04,1.20) | 1 | 1.01(0.91,1.12) | 1.07(0.97,1.18) | 1.14(1.03,1.26) | <0.001 |  |
| **Physical activity (MET-minutes/week)** |  |  |  |  |  |  | 0.47 |
| <600 | 1.06(1.00,1.13) | 1 | 0.95(0.88,1.03) | 0.99(0.91,1.07) | 1.03(0.95,1.12) | 0.05 |  |
| 600-3000 | 1.08(1.05,1.13) | 1 | 1.02(0.97,1.07) | 1.06(1.01,1.11) | 1.08(1.03,1.14) | <0.001 |  |
| >3000 | 1.05(1.01,1.10) | 1 | 0.99(0.94,1.05) | 0.97(0.92,1.03) | 1.07(1.01,1.13) | <0.001 |  |
| **BMI (kg/m²)** |  |  |  |  |  |  | 0.50 |
| <25 | 1.09(1.05,1.13) | 1 | 0.98(0.93,1.04) | 1.02(0.97,1.08) | 1.11(1.05,1.17) | <0.001 |  |
| ≥25 | 1.06(1.03,1.09) | 1 | 0.99(0.96,1.03) | 1.00(0.97,1.04) | 1.05(1.01,1.09) | <0.001 |  |
| **Hypertension** |  |  |  |  |  |  | 0.64 |
| Yes | 1.08(1.05,1.12) | 1 | 0.98(0.94,1.03) | 1.01(0.97,1.06) | 1.07(1.03,1.12) | <0.001 |  |
| No | 1.06(1.03,1.10) | 1 | 1.00(0.96,1.04) | 1.00(0.96,1.05) | 1.07(1.02,1.11) | <0.001 |  |
| **Diabetes** |  |  |  |  |  |  | 0.04 |
| Yes | 1.17(1.09,1.25) | 1 | 1.11(1.01,1.23) | 1.15(1.04,1.27) | 1.25(1.13,1.37) | <0.001 |  |
| No | 1.05(1.01,1.09) | 1 | 0.98(0.95,1.01) | 1.00(0.97,1.03) | 1.06(1.02,1.09) | <0.001 |  |
| **CKM stage** |  |  |  |  |  |  | 0.47 |
| 0 | 1.06(1.01,1.12) | 1 | 0.96(0.94,1.02) | 1.02(1.00,1.05) | 1.09(1.07,1.12) | <0.001 |  |
| 1 | 1.07(1.04,1.13) | 1 | 0.99(0.96,1.04) | 1.03(1.00,1.11) | 1.12(1.06,1.15) | <0.001 |  |
| 2 | 1.11(1.08,1.15) | 1 | 1.04(1.01,1.08) | 1.09(1.04,1.13) | 1.16(1.08,1.20) | <0.001 |  |
| 3 | 1.14(1.11,1.18) | 1 | 1.06(1.04,1.12) | 1.07(1.02,1.10) | 1.18(1.09,1.24) | <0.001 |  |

Analyses were based on Model 3 with comprehensive adjustments, including age, sex, race, Townsend Deprivation Index, education level, alcohol consumption, smoking status, sleep duration, physical activity, diabetes, hypertension, BMI, LDL-C, HDL-C, and hs-CRP.

**Supplementary Table 6. Subgroup analyses of the association of SIRI with incidence of CVD in individuals with CKM syndrome**

| **SIRI** | **Continuous** | **Q1** | **Q2** | **Q3** | **Q4** | **P for trend** | **P for interaction** |
| --- | --- | --- | --- | --- | --- | --- | --- |
| **Age (years)** |  |  |  |  |  |  | 0.44 |
| <60 | 1.26(1.19,1.34) | 1 | 1.05(1.00,1.10) | 1.12(1.07,1.17) | 1.17(1.11,1.22) | <0.001 |  |
| ≥60 | 1.36(1.30,1.43) | 1 | 1.05(1.01,1.10) | 1.10(1.06,1.15) | 1.21(1.16,1.26) | <0.001 |  |
| **Sex** |  |  |  |  |  |  | 0.72 |
| male | 1.32(1.26,1.39) | 1 | 1.03(0.99,1.08) | 1.09(1.04,1.14) | 1.19(1.14,1.24) | <0.001 |  |
| female | 1.34(1.26,1.43) | 1 | 1.07(1.02,1.12) | 1.13(1.08,1.18) | 1.18(1.13,1.24) | <0.001 |  |
| **Race** |  |  |  |  |  |  | 0.61 |
| White | 1.57(1.51,1.63) | 1 | 1.09(1.06,1.13) | 1.20(1.16,1.24) | 1.36(1.32,1.40) | <0.001 |  |
| Non-White | 1.67(1.38,2.02) | 1 | 1.15(1.01,1.31) | 1.20(1.04,1.38) | 1.35(1.16,1.57) | <0.001 |  |
| **Education** |  |  |  |  |  |  | 0.53 |
| College/university degree | 1.48(1.39,1.57) | 1 | 1.06(1.01,1.12) | 1.19(1.14,1.25) | 1.29(1.23,1.35) | <0.001 |  |
| Other qualification | 1.65(1.54,1.78) | 1 | 1.12(1.05,1.19) | 1.17(1.12,1.26) | 1.39(1.31,1.48) | <0.001 |  |
| No qualification | 1.66(1.50,1.83) | 1 | 1.12(1.03,1.23) | 1.29(1.18,1.40) | 1.44(1.32,1.56) | <0.001 |  |
| **Smoke** |  |  |  |  |  |  | 0.49 |
| Current | 1.25(1.14,1.37) | 1 | 1.02(0.93,1.12) | 1.12(1.02,1.22) | 1.18(1.09,1.29) | <0.001 |  |
| Former | 1.36(1.27,1.45) | 1 | 1.12(0.97,1.08) | 1.06(1.01,1.12) | 1.18(1.12,1.24) | <0.001 |  |
| Never | 1.20(1.14,1.27) | 1 | 1.06(1.01,1.11) | 1.10(1.06,1.15) | 1.12(1.07,1.17) | <0.001 |  |
| **Alcohol consumption** |  |  |  |  |  |  | 0.15 |
| Daily | 1.30(1.20,1.42) | 1 | 1.03(0.96,1.10) | 1.08(1.01,1.15) | 1.17(1.10,1.25) | <0.001 |  |
| 3-4 times/week | 1.40(1.29,1.52) | 1 | 1.03(0.96,1.10) | 1.10(1.03,1.17) | 1.20(1.12,1.28) | <0.001 |  |
| 1-2 times/week | 1.24(1.14,1.34) | 1 | 1.04(0.98,1.11) | 1.10(1.04,1.18) | 1.14(1.07,1.22) | <0.001 |  |
| 1-3 times/month | 1.21(1.07,1.37) | 1 | 1.11(1.00,1.22) | 1.15(1.04,1.27) | 1.13(1.03,1.25) | <0.001 |  |
| Special occasions | 1.49(1.33,1.66) | 1 | 1.10(1.00,1.21) | 1.21(1.10,1.32) | 1.33(1.21,1.45) | <0.001 |  |
| Never | 1.25(1.10,1.42) | 1 | 1.08(0.97,1.21) | 1.03(0.92,1.14) | 1.20(1.08,1.33) | <0.001 |  |
| **Physical activity (MET-minutes/week)** |  |  |  |  |  |  | 0.28 |
| <600 | 1.26(1.14,1.40) | 1 | 1.00(0.92,1.10) | 1.07(0.98,1.17) | 1.10(1.02,1.20) | <0.001 |  |
| 600-3000 | 1.31(1.23,1.40) | 1 | 1.06(1.00,1.11) | 1.11(1.05,1.17) | 1.18(1.12,1.24) | <0.001 |  |
| >3000 | 1.32(1.22,1.43) | 1 | 1.06(1.00,1.13) | 1.09(1.02,1.15) | 1.21(1.14,1.28) | <0.001 |  |
| **BMI (kg/m²)** |  |  |  |  |  |  | 0.68 |
| <25 | 1.37(1.28,1.47) | 1 | 1.10(0.95,1.06) | 1.08(1.02,1.14) | 1.21(1.14,1.27) | <0.001 |  |
| ≥25 | 1.27(1.22,1.34) | 1 | 1.08(1.04,1.12) | 1.12(1.08,1.16) | 1.17(1.13,1.22) | <0.001 |  |
| **Hypertension** |  |  |  |  |  |  | 0.49 |
| Yes | 1.34(1.27,1.41) | 1 | 1.05(1.01,1.10) | 1.12(1.07,1.17) | 1.21(1.16,1.26) | <0.001 |  |
| No | 1.28(1.21,1.36) | 1 | 1.05(1.01,1.10) | 1.10(1.05,1.15) | 1.16(1.11,1.21) | <0.001 |  |
| **Diabetes** |  |  |  |  |  |  | <0.01 |
| Yes | 1.43(1.26,1.61) | 1 | 1.09(0.97,1.21) | 1.15(1.03,1.28) | 1.30(1.17,1.44) | <0.001 |  |
| No | 1.20(1.15,1.36) | 1 | 1.05(1.02,1.09) | 1.10(1.07,1.14) | 1.18(1.14,1.21) | <0.001 |  |
| **CKM stage** |  |  |  |  |  |  | 0.58 |
| 0 | 1.22(1.10,1.29) | 1 | 1.05(0.99,1.12) | 1.10(1.06,1.15) | 1.13(1.10,1.20) | <0.001 |  |
| 1 | 1.28(1.14,1.40) | 1 | 1.07(1.04,1.10) | 1.12(1.08,1.15) | 1.16(1.12,1.20) | <0.001 |  |
| 2 | 1.39(1.23,1.40) | 1 | 1.11(1.08,1.15) | 1.18(1.15,1.20) | 1.20(1.14,1.25) | <0.001 |  |
| 3 | 1.49(1.33,1.66) | 1 | 1.13(1.10,1.16) | 1.21(1.18,1.26) | 1.34(1.22,1.46) | <0.001 |  |

Analyses were based on Model 3 with comprehensive adjustments, including age, sex, race, Townsend Deprivation Index, education level, alcohol consumption, smoking status, sleep duration, physical activity, diabetes, hypertension, BMI, LDL-C, HDL-C, and hs-CRP.

**Supplementary Table 7. Sensitivity analysis on exclusion of participants with follow-up less than 2 years**

| **CVD** | **Model 1** | **Model 2** | **Model 3** |
| --- | --- | --- | --- |
| **SII** |  |  |  |
| Continuous | 1.10(1.07,1.12) | 1.10(1.06,1.11) | 1.08(1.06,1.11) |
| Categories |  |  |  |
| Q1 | 1 | 1 | 1 |
| Q2 | 0.99(0.96,1.02) | 1.00(0.97,1.02) | 1.00(0.95,1.02) |
| Q3 | 1.02(0.99,1.06) | 1.03(1.00,1.06) | 1.01(0.98,1.05) |
| Q4 | 1.10(1.07,1.14) | 1.09(1.07,1.13) | 1.07(1.04,1.10) |
| p-trend | <0.001 | <0.001 | <0.001 |
| **SIRI** |  |  |  |
| Continuous | 1.42(1.36,1.47) | 1.41(1.35,1.46) | 1.29(1.24,1.35) |
| Categories |  |  |  |
| Q1 | 1 | 1 | 1 |
| Q2 | 1.07(1.04,1.11) | 1.08(1.04,1.11) | 1.06(1.02,1.09) |
| Q3 | 1.15(1.11,1.19) | 1.14(1.12,1.19) | 1.11(1.08,1.15) |
| Q4 | 1.26(1.23,1.30) | 1.25(1.22,1.30) | 1.18(1.14,1.22) |
| p-trend | <0.001 | <0.001 | <0.001 |

Model 1 adjusted for age and sex; Model 2 additionally included ethnicity, socioeconomic status, education, alcohol use, smoking, sleep status, and physical activity; and Model 3 further adjusted for diabetes, hypertension, BMI, LDL-C, HDL-C, eGFR, and hs-CRP.

**Supplementary Table 8. Sensitivity analysis of PSM to balance covariates**

| **CVD** | **Model 1** | **Model 2** | **Model 3** |
| --- | --- | --- | --- |
| **SII** |  |  |  |
| Continuous | 1.10(1.07,1.12) | 1.11(1.07,1.12) | 1.07(1.05,1.10) |
| Categories |  |  |  |
| Q1 | 1 | 1 | 1 |
| Q2 | 0.99(0.96,1.02) | 0.98(0.96,1.02) | 0.99(0.95,1.02) |
| Q3 | 1.02(0.99,1.05) | 1.02(0.98,1.05) | 1.01(0.98,1.03) |
| Q4 | 1.10(1.07,1.14) | 1.10(1.07,1.13) | 1.07(1.04,1.10) |
| p-trend | <0.001 | <0.001 | <0.001 |
| **SIRI** |  |  |  |
| Continuous | 1.43(1.38,1.49) | 1.42(1.37,1.48) | 1.31(1.26,1.36) |
| Categories |  |  |  |
| Q1 | 1 | 1 | 1 |
| Q2 | 1.07(1.03,1.10) | 1.07(1.04,1.11) | 1.05(1.02,1.09) |
| Q3 | 1.14(1.11,1.18) | 1.15(1.11,1.18) | 1.11(1.07,1.14) |
| Q4 | 1.27(1.23,1.30) | 1.26(1.22,1.30) | 1.19(1.15,1.22) |
| p-trend | <0.001 | <0.001 | <0.001 |

Model 1 adjusted for age and sex; Model 2 additionally included ethnicity, socioeconomic status, education, alcohol use, smoking, sleep status, and physical activity; and Model 3 further adjusted for diabetes, hypertension, BMI, LDL-C, HDL-C, eGFR, and hs-CRP.

**Supplementary Table 9. Sensitivity analysis of Age as the timescale**

| **CVD** | **Model 1** | **Model 2** | **Model 3** |
| --- | --- | --- | --- |
| **SII** |  |  |  |
| Continuous | 1.11(1.08,1.12) | 1.10(1.07,1.12) | 1.07(1.05,1.09) |
| Categories |  |  |  |
| Q1 | 1 | 1 | 1 |
| Q2 | 1.00(0.96,1.02) | 0.99(0.96,1.02) | 0.99(0.96,1.02) |
| Q3 | 1.03(1.00,1.05) | 1.02(0.99,1.05) | 1.01(0.98,1.04) |
| Q4 | 1.11(1.07,1.14) | 1.10(1.07,1.13) | 1.07(1.04,1.10) |
| p-trend | <0.001 | <0.001 | <0.001 |
| **SIRI** |  |  |  |
| Continuous | 1.46(1.41,1.52) | 1.45(1.40,1.50) | 1.33(1.28,1.39) |
| Categories |  |  |  |
| Q1 | 1 | 1 | 1 |
| Q2 | 1.07(1.04,1.11) | 1.08(1.04,1.11) | 1.06(1.03,1.09) |
| Q3 | 1.15(1.12,1.19) | 1.16(1.12,1.19) | 1.12(1.08,1.15) |
| Q4 | 1.28(1.24,1.32) | 1.26(1.23,1.32) | 1.20(1.16,1.24) |
| p-trend | <0.001 | <0.001 | <0.001 |

Model 1 adjusted for age and sex; Model 2 additionally included ethnicity, socioeconomic status, education, alcohol use, smoking, sleep status, and physical activity; and Model 3 further adjusted for diabetes, hypertension, BMI, LDL-C, HDL-C, eGFR, and hs-CRP.

**Supplementary Table 10. Sensitivity analysis excluding CKM-defining variables**

| **CVD** | **Model (excluding CKM-defining variables)** |
| --- | --- |
| **SII** |  |
| Continuous | 1.08(1.06,1.11) |
| Categories |  |
| Q1 | 1 |
| Q2 | 1.01(0.98,1.04) |
| Q3 | 1.03(1.01,1.06) |
| Q4 | 1.08(1.05,1.12) |
| p-trend | <0.001 |
| **SIRI** |  |
| Continuous | 1.33(1.29,1.39) |
| Categories |  |
| Q1 | 1 |
| Q2 | 1.07(1.03,1.10) |
| Q3 | 1.13(1.10,1.17) |
| Q4 | 1.21(1.17,1.24) |
| p-trend | <0.001 |

Model (excluding CKM-defining variables) adjusted for age, sex, ethnicity, socioeconomic status, education, alcohol use, smoking status, sleep status, physical activity, and hs-CRP.
